# A clustering approach for topic filtering within systematic literature reviews

**DOI:** 10.1016/j.mex.2020.100831

**Published:** 2020-02-22

**Authors:** Tim Weißer, Till Saßmannshausen, Dennis Ohrndorf, Peter Burggräf, Johannes Wagner

**Affiliations:** Chair for International Production Engineering and Management, University of Siegen

**Keywords:** Systematic literature review, Literature filtering, Clustering

## Abstract

Within a systematic literature review (SLR), researchers are confronted with vast amounts of articles from scientific databases, which have to be manually evaluated regarding their relevance for a certain field of observation. The evaluation and filtering phase of prevalent SLR methodologies is therefore time consuming and hardly expressible to the intended audience. The proposed method applies natural language processing (NLP) on article meta data and a k-means clustering algorithm to automatically convert large article corpora into a distribution of focal topics. This allows efficient filtering as well as objectifying the process through the discussion of the clustering results. Beyond that, it allows to quickly identify scientific communities and therefore provides an iterative perspective for the so far linear SLR methodology.

• NLP and k-means clustering to filter large article corpora during systematic literature reviews.
• Automated clustering allows filtering very efficiently as well as effectively compared to manual selection.
• Presentation and discussion of the clustering results helps to objectify the nontransparent filtering step in systematic literature reviews.

## GRAPHICAL ABSTRACT

**Figure:**
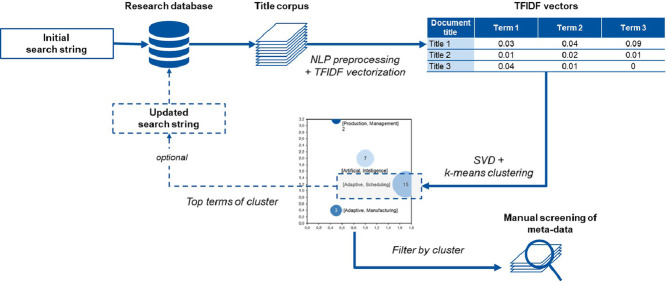

**Table:** Specification Table

Subject Area:	*Social Sciences*
More specific subject area:	*Literature analysis*
Method name:	*Systematic Literature Review (SLR)*
Name and reference of original method:	• Brocke et al. (2009) - Reconstructing the giant: on the rigor in documenting the literature search process • Moher et al. [15] - Preferred Reporting Items for Systematic Reviews and Meta-Analyses
Resource availability:	*If applicable, include links to resources necessary to reproduce the method (*e.g. *data, software, hardware, reagent)*

## Method details

Every scientific examination of a new topic begins with a research on the current state of the art. The latter is reflected in latest publications, wherefore a literature research is often the first method of choice. To meet scientific standards, the method of choice is usually a systematic literature review (SLR). Depending on its objective, different typologies of SLR can be chosen for this purpose [16]. Thus, the search types ``narrative'', ``descriptive'' and ``scoping'' focus on the objective of *summarizing existing knowledge* [16]. Further objectives are *data aggregation, explanation formation* and *critical evaluation* [16]. Parallel to these different typologies and objectives, there are a number of authors who describe different procedural models. First, these models vary in their range: with starting points ranging from "definition of review scope" [6] to "database search" [15] and end points from "definition of electronic sources" [5] to "set up of research agenda" [6]. Second, just like the range of the various procedural models, the level of detail and thus the number of phases differ between the authors. Boland et al. [4] and Fink [8] for example came along with an approach of ten phases each. In contrast, the approach of Levy & Ellis [14] consists of only three phases. To summarize, different authors have different objectives when describing the approach of SLR: While some describe the overall approach in rough terms (e.g. [6]), other authors focus on specific phases and explain them in detail (e.g. [15]). For a comparison of these different procedural models, see section *supplementary material*, Table 2.

However, all these methodologies have in common that they contain the phases *literature search* and *literature analysis*. The literature search comprises the systematic search with a defined search string in defined databases (e.g. [5]). In the phase of literature analysis, the results of the search are evaluated in order to filter out irrelevant articles [15]. The search and the analysis are regarded exclusively as linear procedural phases (e.g. [8]). Although Brocke et al. [6] describe the overall process of SLR as iterative, from the definition of the research focus to a new research agenda, the literature search and literature analysis phases also build on each other linearly. In addition to this criticism of linearity, there is also a lack of efficient methods to ensure objectivity in evaluation and filtering the articles during the phase of literature analysis. The current way to ensure objectivity is to carry out the screening of titles and abstracts, the reading of full texts and the respective evaluation by several independent reviewers [8]. Even if filtered by several reviewers, the evaluation criteria for the exclusion or retention of a paper are usually not fully transparent - in the worst case they remain a black box.

## Procedure

The aim of this paper is to address the problems of linearity as well as inefficiency and lack of transparency in the phases of literature research and analysis by extending the existing methods. We therefore apply a clustering algorithm on article titles, keywords and abstracts that allows us to automatically break down large article corpora into distinct topical groups. This methodological extension ensures on the one hand the iterative adaptation of the search string in the literature search phase and on the other hand the efficient, objective, and transparent filtering of articles in the literature analysis phase.

### Data acquisition

A lot of meta data from research articles/documents indexed in the biggest scientific databases is freely accessible via search engines of different publishing companies (cf. IEEE, Emerald Insight, MEDLINE…). The amount of retrieved meta data varies by company, but in most cases comprises a set of authors, year, title, keywords and abstract. Furthermore, contemporary search engines allow exporting the data to different file formats such as BibTeX, xml and csv. As in our approach, those are readable via Python standard libraries. If necessary, freeware solutions allow a conversion of not relational formats, such as BibTeX.

### Natural language processing-pipeline

In order to analyze unstructured data, such as text, as well as to achieve an adequate distribution by means of a mathematical clustering model, the following natural language preprocessing (NLP) pipeline is used on meta data of the collection of articles:

1. Removal of duplicates (as articles can be indexed in several databases)
2. Tokenization (word separation)
3. Stop word and punctuation removal (as stop words do not contain relevant information)
4. Optional: Search term removal (as those are already familiar and affect clustering results)
5. Language detection to filter non-English results
6. Optional: Mapping of synonyms and hypernyms
7. Optional: Lemmatization/stemming (back-spacing of verbs and adjectives to a basic form to raise comparability)
8. TFIDF vectorization (ratio of term frequency and inverse document frequency), to consider relative term relevance

The optional search term removal is integrated into the NLP pipeline because words from the initial search term may bias the clustering results as they already form specific communities. In this case, we want to only focus on communities (e.g. clusters) from outside of aforementioned predefined groupings. Therefore, search terms are removed from the vocabulary of the text corpus entirely, similar to stop words removal. The last step in the NLP pipeline is the TFIDF vectorization, therefore the TFIDF measure is calculated via

(1) $$TFIDF\left(w\right)=\;c\left(w\right)\cdot log\frac{D}{d\left(w\right)}$$

As TFIDF weighs the number of term occurrences *c*(*w*) in a certain document against the occurrences of that term within the corpus of all documents *d*(*w*), relative to the total number of documents *D*, it is a good indicator for the relevance of a term [2]. For larger text proportions, for example if clustering is intended to be used on abstracts or full texts, word embeddings are recommended. The output of the NLP pipeline is a set of vectors, each representing a title in the corpus (respectively keywords or abstract) with the vector length being the amount of words in the whole corpus. This document-term matrix with TFIDF decimals is suitable for mathematical processing and analysis.

### Latent semantic analysis (LSA)

Natural language offers a high degree of freedom, often resulting in noisy data. This leads to diffuse clustering results because of inflating Euclidean distances between data points (see *curse of dimensionality* by [3]). This problem gets worse with increasing corpus size as the number of features increases with more text. Thus, an LSA is applied for a prior dimensionality reduction (as proposed in [2] as well as in [1]), by performing a singular value decomposition (SVD) on the document-term matrix. Through this, the term-document matrix is decomposed into a multiplication of three eigenvalue matrices representing the initial matrix. The resulting matrix diagonally contains scaling eigenvalues of which the smallest values are omitted up to a degree *k*. Hereby the dimensionality is reduced and only those concepts remain that are semantically most relevant. In the presented approach, it is specified by default that the number of principal components should explain at least 30% of the variance of the data set. This results in the number of components *k* for further calculations determined by

(2) $$\frac{\sum_{i=1}^{k}\sigma_{i}}{\sum_{j=1}^{r}\sigma_{j}}\geq 0.30$$

with *r* being the number of original components and *σ* being each of their variance [7,^12^,13]. The optimal number of remaining components depends on the corpus’ extent, as well as the intended number of clusters.

### Clustering

To simplify and objectify the literature evaluation, the actual analysis of the corpus is achieved through structuring/sorting the data according to comprehensive groups of similar concepts, methods and technologies. Clustering algorithms are sensitive to i.a. corpus size, document size, and linguistic precision of the domain, which is why the clustering quality strongly depends on the assessment of the domain expert [10]. It can be meaningful to apply different algorithms depending on the data. Both, partitional clustering such as k-means as well as agglomerative hierarchical clustering methods achieve comparable results for document clustering [11]. With regards to large corpora in the use case of an SLR, the grouping is obtained by applying a k-means algorithm, as it offers both, excellent time complexity and scalability as well as a good cluster purity [17]. The aim of the k-means algorithm is to divide a given number of samples into a predefined set *C* of *K* clusters by minimizing the sum of squared errors (SSE), also called inertia, between data points *x_i_* and the cluster means *μ_k_* as shown in Eq. (3) [9], [10].

(3) $$J\left(C\right)=\sum_{k=1}^{K}\sum_{x_{i}\in c_{k}}{∥x_{i}-\mu_{k}∥}^{2}$$

The SSE measure is also utilizable to determine an appropriate number of clusters. By plotting the SSE of several algorithm runs with different *k*, a distinctive flattening of the curve can often be observed at a certain *k* (named elbow). To achieve good clustering results, the value for *k* should be chosen as little as possible and as large as necessary. This optimal number of *k* is found at the elbow kink. To evaluate the clustering quality, it is also suitable to calculate the silhouette score (SSC). The SSC compares the distance of a data point to data points of its assigned cluster with the distances to data points of the neighboring clusters: Be *a*(*i*) the average dissimilarity of an object *i* to all other objects in its own cluster *A* (representing the second best choice for *i*, as well as *b*(*i*) as the average dissimilarity of *i* to objects in the closest cluster to *A*. The SSC can then be calculated as follows [18]:

(4) $$s\left(i\right)=\;\frac{b\left(i\right)\;-\;a\left(i\right)}{max\left\{a\left(i\right)\;-\;b\left(i\right)\right\}}$$

### Interpretation

To interpret the clustering results, we extract and display the most relevant words of every cluster (according to their TFIDF value in the cluster's centroid), as well as the cluster size (by number of assigned samples), the cluster's centroid and its most centric document according to the Euclidean distance to the centroid. These metrics allow for findings concerning the most prevalent concepts, methods and technologies, associated with the initially used search terms. Furthermore, significant research communities are identifiable, as well as the attention of topics and solutions. The clustering approach within the literature filtering stage of an SLR is hence:

– efficient and reusable through the automated analysis of large corpora
– objectified by presenting the conclusions of what to include and exclude for further analysis within the SLR

Possible adaptions of the clustering method include the use of keywords or abstracts instead of only titles. In this regard, results show that clustering based on keywords is reasonable for larger corpora, as authors tend to use common descriptors in them, which simplifies clustering. Contrarily and for smaller corpora, the depth of analysis can be increased by including abstracts into the clustering.

### Exploring topics

**Fig. 1:**
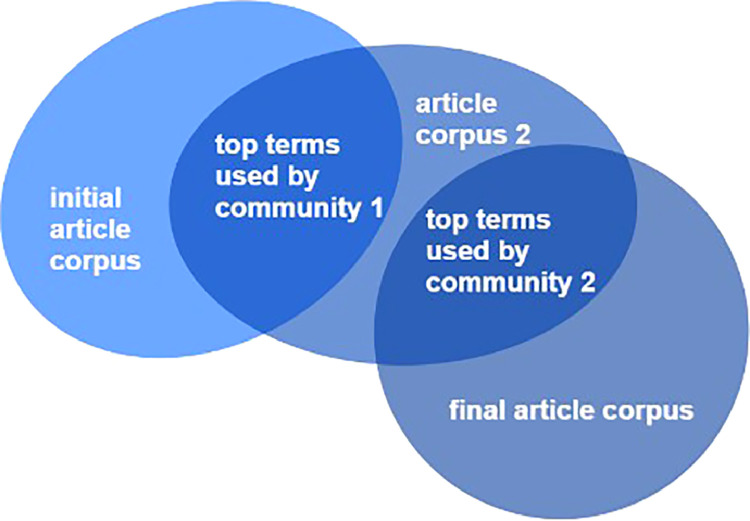
Finding communities via iterative search and clustering.

In an SLR, the main problem is often to find a community that is tailored to the topic in question. Therefore, an approach is presented, that can find very specific communities by iteratively adjusting and sharpening the search string. First, a generic search string can be used, which roughly points in the corresponding direction. An example would be the search string *Production AND Machine Learning*. Subsequently, corresponding articles are searched in the above-mentioned databases, creating an initial rough article corpus. In this corpus, the first step is to further filter communities by the proposed clustering procedure. After that, the relevant communities’ top terms are extracted, which in turn defines a new search string that leads to a new corpus of articles via iterative search in the corresponding databases. This corpus is then again redirected through the NLP pipeline as well as clustering so that new communities and their specific top terms are captured which could not previously be captured using the initial search term. This process is repeated until the community has been severely restricted (see Fig. 1) and the final article corpus is found. The proposed procedure can also be used exploratory to identify the search term for an SLR in the first place.

## Validation

For validation the generic search string *(Production OR Manufacturing) AND (Artificial Intelligence OR Machine Learning)* is used, as it covers a broad scope and shows specifically how the proposed methodology can deal with unspecific and diverse text corpora. The clustering pipeline which is based on the beforementioned NLP pipeline for preprocessing is specified in Algorithm 1 in the supplementary material. Algorithm 1 was applied for the validation process and its notation is based on [2]. The initial search in the four different databases *IEEE, ACM, Web of Science* and *ScienceDirect* returned 521 articles. Searches were only performed in the *title* field. The results were preprocessed according to the proposed NLP pipeline. After deletion of duplicates and artifacts, the text corpus carries 291 unique documents on which tokenization, stop word removal, stemming and TFIDF vectorization were performed. These actions were performed on the document title, the keywords and the abstract respectively. The resulting word vectors were reduced to their principal components using SVD, considering a fixed minimum explained variance of 30%. This minimum explained variance is achieved for abstracts, keywords and titles according to the number of components Table 3 of the supplementary material.

**Algorithm 1:** The proposed clustering algorithm used for validation (according to [2]).

**Fig. 2:**
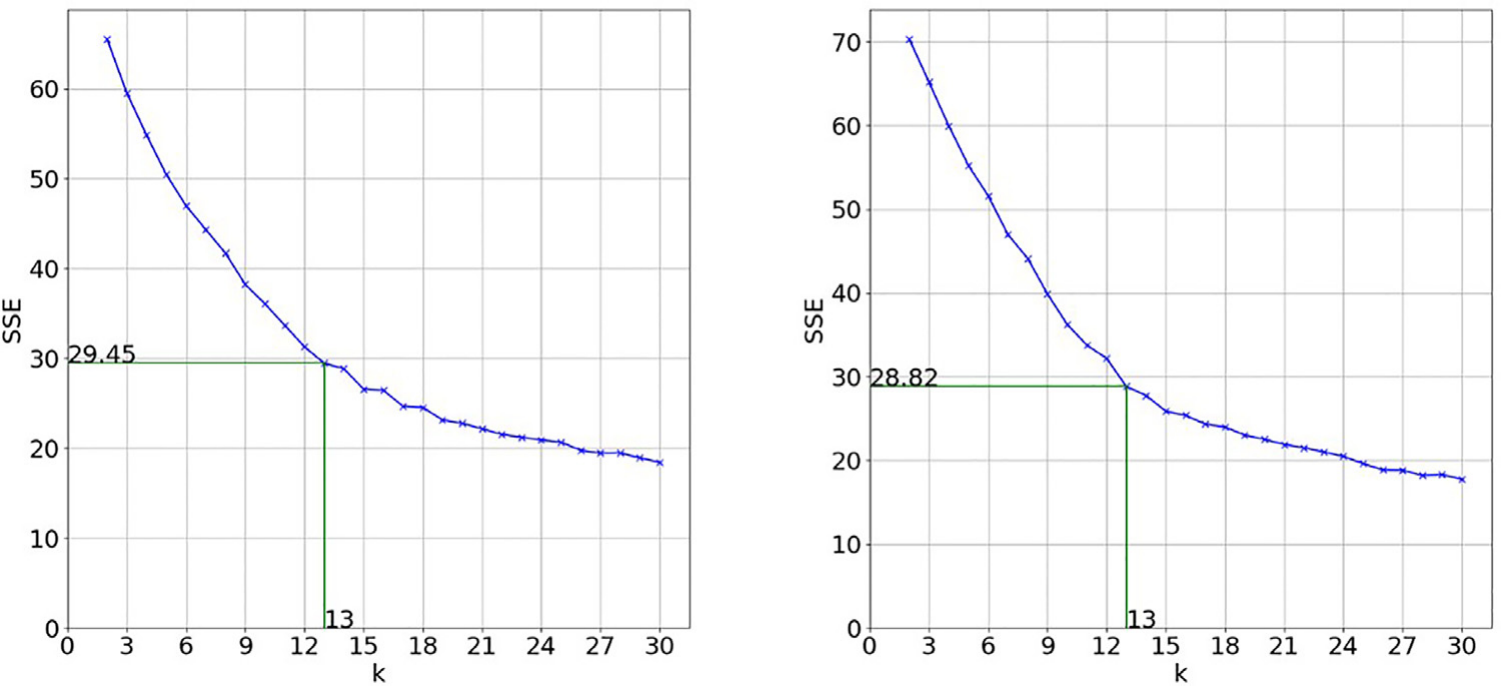
SSE for title with search string (left) and SSE for title without search string (right).

**Fig. 3:**
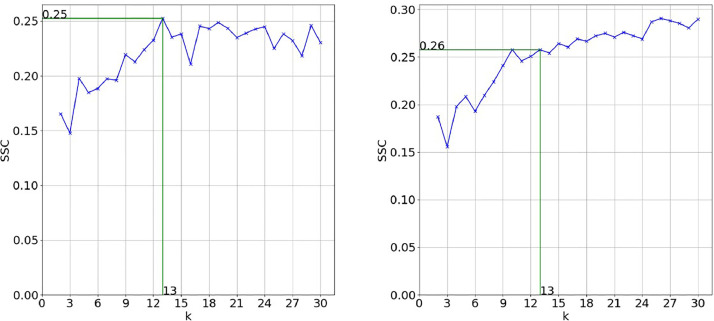
SSC for title with search string (left) and SSC for title without search string (right).

**Fig. 4:**
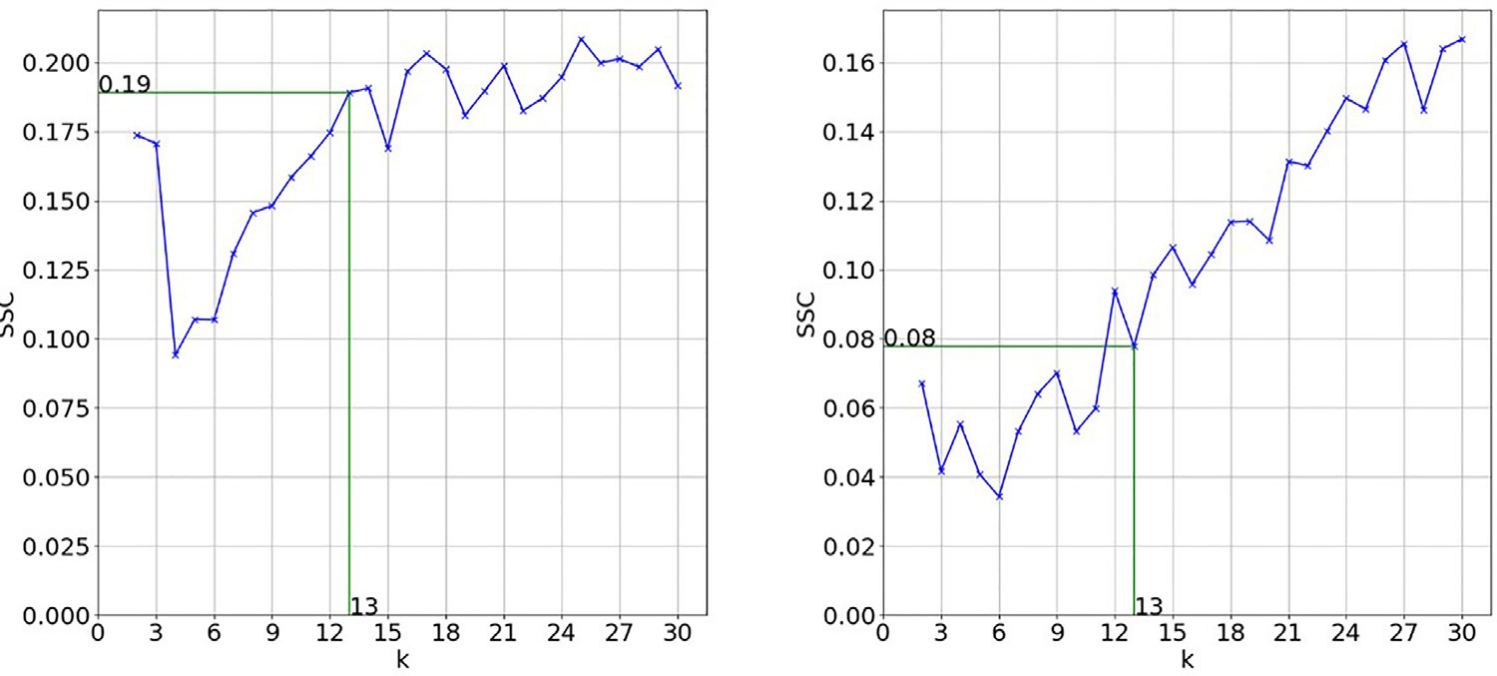
SSC for Keywords without search string (left) and SSC for abstract without search string (right).

To get the optimal number of clusters, the silhouette score (SSC) was combined with the elbow method. Fig. 2 shows the evolution of the SSE on which the elbow method is based. For convenience of further interpretation in the literature analysis process, the maximum number of clusters to be considered for evaluation is manually set to 30. In this case, the document title achieved the best results with an elbow at k=13clusters. The deletion of the initial search string from the vocabulary (Fig. 2) does not change the results significantly. The silhouette scores (Fig. 3) confirm these observations. For keywords and abstracts, the evaluation metrics show inferior results (see Fig. 4). One possible explanation is that the text extracted from abstracts is too complex and suffers from the curse of dimensionality to a bigger extent than the title so that even with dimensionality reduction there can be seen no clear structure. So, with a minimum variance of 30%, the best results are achieved by doing the clustering based on the titles of documents.

**Fig. 5:**
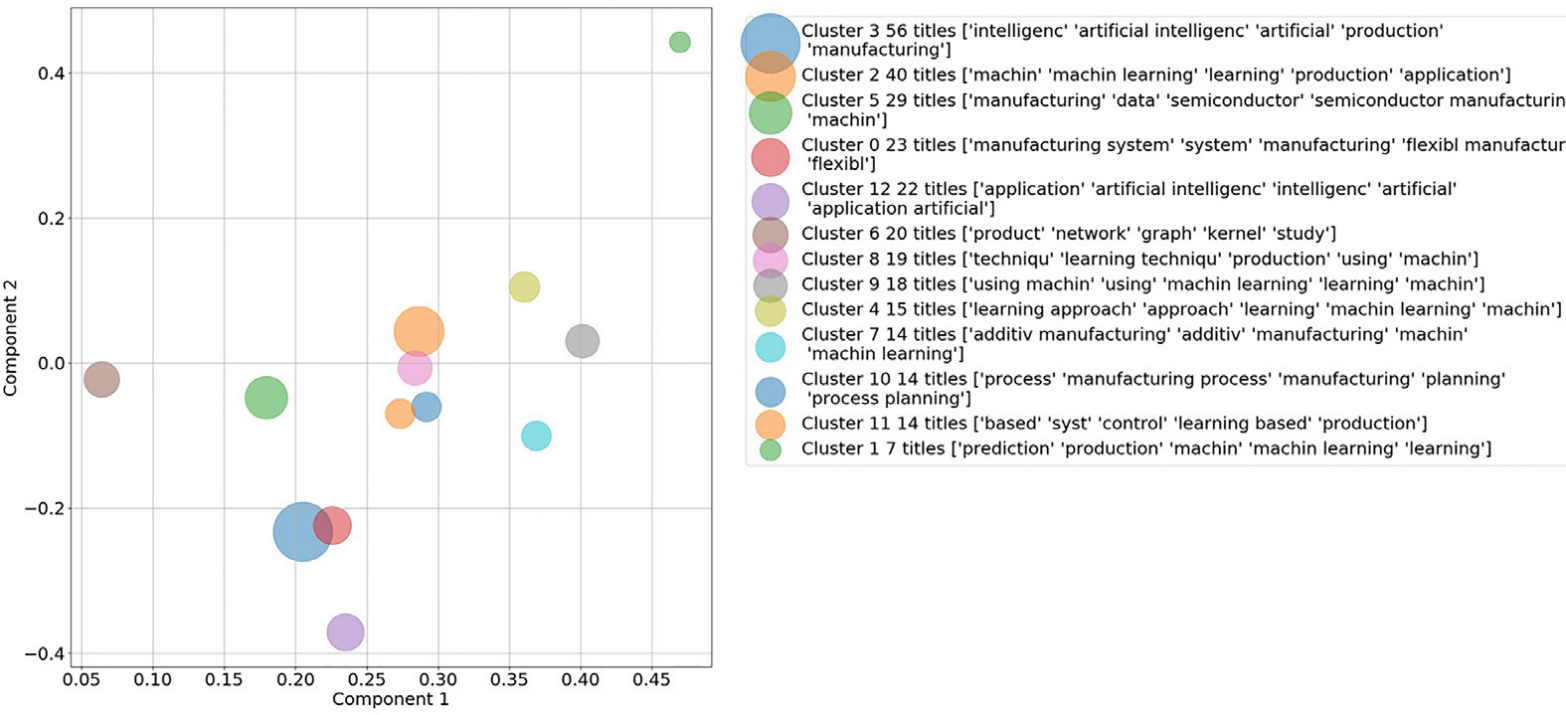
Two-dimensional representation of generated clusters and their top words based on document titles for *k* = 13 with search string included in vocabulary.

As for a comparison between deleting and keeping the search string, the 13 clusters and their top words are evaluated in detail for the document title in Fig. 5 and Fig. 6 in the supplementary material. Based on the clusters’ top words and the achieved SSE and SSC in Fig. 2 and Fig. 3, the decision was made to delete the search string from the corpus vocabulary and to only consider the document titles with deleted search string for further validation.

**Fig. 6:**
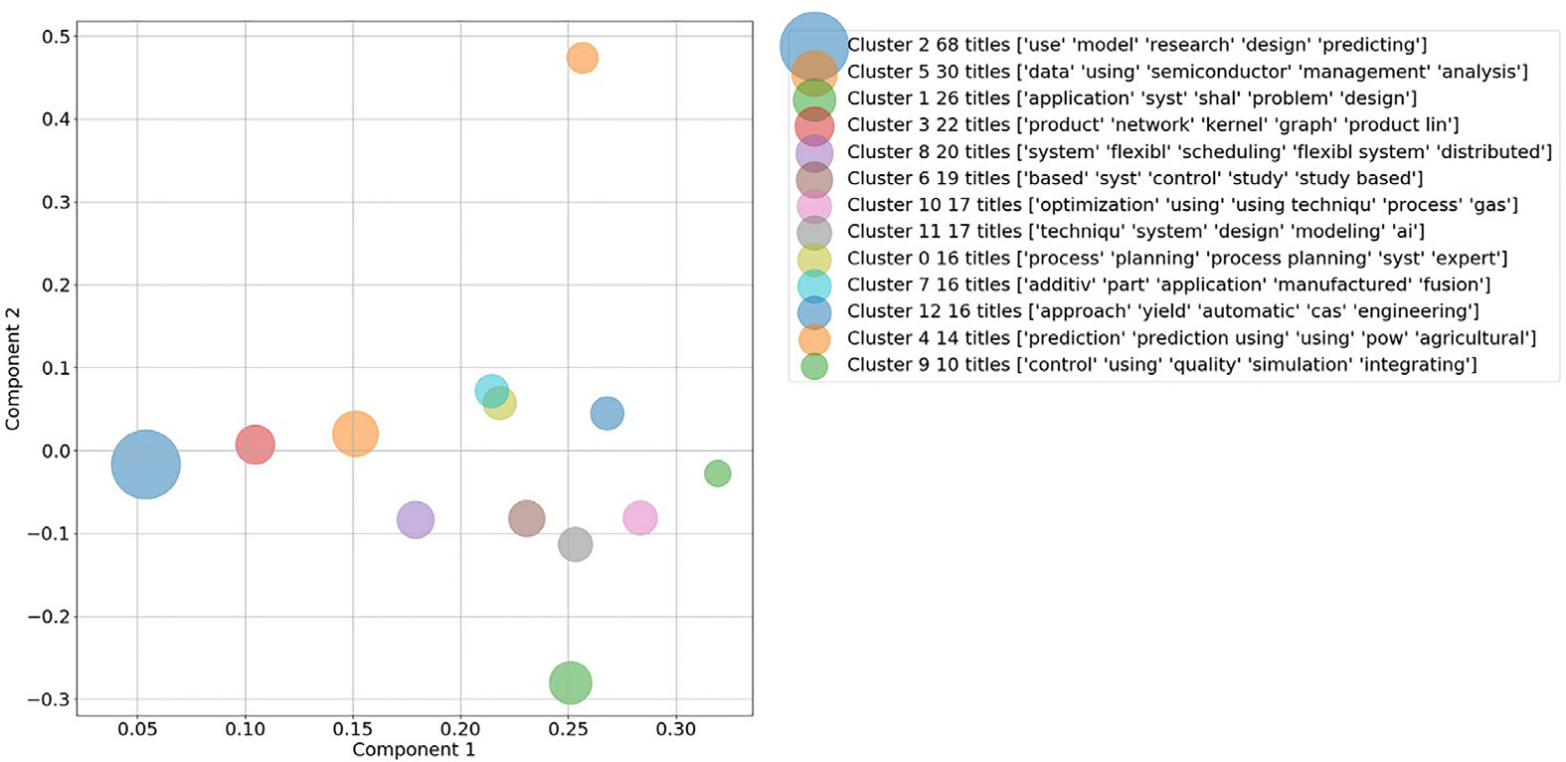
Two-dimensional representation of generated clusters and their top words based on document titles for *k* = 13 with search string excluded from vocabulary.

The result of the k-means clustering based on the SVD corpus is 13 thematically separate topic clusters (see Table 4 and Fig. 6). For evaluating the informative value of the clusters, the average TFIDF score of the top five terms inside each cluster's centroid is calculated. This five-term score is an indicator for the integrity of the cluster as it shows how frequent the top terms in this cluster are compared to the rest of the document corpus. To give a comprehensive overview, the four clusters which were identified as most relevant for the exemplary thematic focus of *Artificial Intelligence and Machine Learning in Production and Manufacturing* are shown in Table 1.

**Table 1:** Most relevant cluster with top terms, cluster size and average TFIDF score.

Cluster No.	Top terms	Cluster size	Avg. TFIDF score
9	*control, using, quality, simulation, integrating*	10	1.1
8	system*, flexibl*, scheduling, flexibl* system*, distributed	20	0.9
7	additiv*, part, application, manufactured, fusion	16	0.8
0	process, planning, process planning, syst*, expert	16	0.9

The validation results show that several distinct groups are identified with the proposed clustering algorithm. These groups form coherent communities: Cluster 9 – *Machine learning for quality control based on simulations*, cluster 8 – *Machine learning in flexible manufacturing scheduling systems*, cluster 7 – *Machine learning in additive manufacturing* and cluster 4 – *Machine learning in production process planning.* For detailed information on the clusters, their centroids and the papers closest to the centroids see Table 4 in the supplementary material.

## Conclusion

First, the described method of text clustering is suitable for oscillating iteratively between the phases of literature research and literature analysis on the basis of a rough search direction and for getting closer to a target research community. In this way, the search string of the SLR is automatically refined by the top terms of clusters of interest, which promises a higher quality of articles for one's own field of interest. Second, the text clustering promotes objectivity, transparency and efficiency in the phase of literature analysis, which is superior to manual analysis, especially for a large number of articles. We identified that especially document titles are suited for such a clustering as they offer a good tradeoff between complexity and information density. It could be shown that abstracts are not that suitable for such big text corpora unless the information is vastly reduced by an SVD with below 30% explained variance. By using the method presented in this work, the task of the reviewer is not to read abstracts and filter articles, but to exclude clusters of articles respectively to integrate clusters of articles of low/high relevance for the topic of interest. Finally, the articles that pass the filtering stage have to be processed according to the successive phases of the SLR procedure. This next step will usually be the phase of full text screening.

## Declaration of Competing Interest

The Authors confirm that there are no conflicts of interest.

## Appendix Supplementary materials

Image, application 1
