# Supplementary material for: A clustering approach for topic filtering within systematic literature reviews
Source: MethodsX. 2020 Feb 22;7:100831. doi: 10.1016/j.mex.2020.100831 (PMC7078380; doi:10.1016/j.mex.2020.100831)
Supplement: Supplementary file 1 [file mmc1.docx]

**Supplementary material**

Table 2: Comparison of different approaches for systematic literature review

| **Levy & Ellis (2006)** | **Brocke et al. (2009)** | **Booth (2006)** | **Moher et al. (2009)** | **Boland et al. (2017)** | **Fink (2019)** |
| --- | --- | --- | --- | --- | --- |
| 1. Input (literature gathering and screening) 2. Processing (iterative)    1. Know the literature    2. Comprehend the literature    3. Apply    4. Analyze    5. Synthesize    6. Evaluate 3. Output (writing the literature review) | 1. Definition of review scope 2. Conceptualization  of topic 3. Literature search 4. Literature analysis and synthesis 5. Research agenda | 1. Sampling strategy 2. Type of studies 3. Approaches 4. Range of years 5. Limits 6. Inclusion and exclusions 7. Terms used 8. Electronic sources | 1. Database search 2. Duplicates removal 3. Screening 4. Full text assessment 5. Qualitative synthesis 6. Quantitative synthesis | 1. Planning your review 2. Scoping searches, identification of review questions 3. Literature search 4. Screening of titles and abstract 5. Obtain articles 6. Selection of full-text articles 7. Data extraction 8. Quality assessment 9. Analysis and synthesis 10. Writing the literature review | 1. Selection of research questions 2. Selection of databases and web sites 3. Selection of search terms (Ask experts to review databases and search terms) 4. Apply practical screen 5. Apply methodological quality screen 6. Train reviewers 7. Pilot test the reviewing process 8. Do the review (monitor quality) 9. Synthesize the results 10. Produce descriptive OR perform meta-analysis |

Table 3: Number of principal components with SVD and given minimum explained variance of 30%

|  | Abstract | | Keywords | | Title | |
| --- | --- | --- | --- | --- | --- | --- |
|  | With string in vocabulary | Without string in vocabulary | With string in vocabulary | Without string in vocabulary | With string in vocabulary | Without string in vocabulary |
| SVD-components | 34 | 35 | 16 | 17 | 13 | 13 |

Table 4: Clusters and their centroids as well as the paper closest to the centroid

| **Cluster** | **Title closest to centroid** | **Euclidean distance to centroid** | **TFIDF-Score (avg.)** | **Cluster Size** |
| --- | --- | --- | --- | --- |
| 0 | Lee, J. H. (2002). Artificial intelligence-based sampling planning system for dynamic manufacturing process. *Expert Systems with Applications*, *22*(2), 117-133. | 0.23 | 0.9 | 16 |
| 1 | Wuest, T., Weimer, D., Irgens, C., & Thoben, K. D. (2016). Machine learning in manufacturing: advantages, challenges, and applications. *Production & Manufacturing Research*, *4*(1), 23-45. | 0.14 | 0.8 | 26 |
| 2 | Voronin, E. A., Kozlov, S. V., & Kubankov, Y. A. (2019, March). Economic Security Assessment of Onboard Equipment Production with Possible Realization in Standard Machine Learning Technologies. In *2019 Systems of Signals Generating and Processing in the Field of on Board Communications* (pp. 1-4). IEEE. | 0.07 | 0.2 | 68 |
| 3 | Hasbi, M., Budiardjo, E. K., & Wibowo, W. C. (2018, December). Reverse Engineering in Software Product Line-A Systematic Literature Review. In *Proceedings of the 2018 2nd International Conference on Computer Science and Artificial Intelligence* (pp. 174-179). ACM. | 0.1 | 0.7 | 22 |
| 4 | Scher, S., & Molinder, J. (2019). Machine Learning-Based Prediction of Icing-Related Wind Power Production Loss. *IEEE Access*, *7*, 129421-129429. | 0.16 | 0.9 | 14 |
| 5 | Kotenko, I., Saenko, I., & Branitskiy, A. (2019). Improving the Performance of Manufacturing Technologies for Advanced Material Processing Using a Big Data and Machine Learning Framework. *Materials Today: Proceedings*, *11*, 380-385. | 0.13 | 0.7 | 30 |
| 6 | Rebouças Filho, P. P., Gomes, S. L., e Nascimento, N. M. M., Medeiros, C. M., Outay, F., & de Albuquerque, V. H. C. (2019). Energy production predication via Internet of Thing based machine learning system. *Future Generation Computer Systems*, *97*, 180-193. | 0.18 | 0.8 | 19 |
| 7 | Douard, A., Grandvallet, C., Pourroy, F., & Vignat, F. (2018, December). An Example of Machine Learning Applied in Additive Manufacturing. In *2018 IEEE International Conference on Industrial Engineering and Engineering Management (IEEM)* (pp. 1746-1750). IEEE. | 0.14 | 0.8 | 16 |
| 8 | Bel, G., & Bensana, E. (1996). Artificial intelligence and job shop scheduling systems in aeronautical manufacturing. *Recherche Aerospatiale*, (1), 11-22. | 0.15 | 0.9 | 20 |
| 9 | Bergmann, S., Feldkamp, N., & Strassburger, S. (2017). Emulation of control strategies through machine learning in manufacturing simulations. *Journal of Simulation*, *11*(1), 38-50. | 0.26 | 1.1 | 10 |
| 10 | Burrows, E. H., Wong, W. K., Fern, X., Chaplen, F. W., & Ely, R. L. (2009). Optimization of ph and nitrogen for enhanced hydrogen production by synechocystis sp. pcc 6803 via statistical and machine learning methods. *Biotechnology Progress*, *25*(4), 1009-1017. | 0.22 | 0.9 | 17 |
| 11 | Monostori, L. (2003). AI and machine learning techniques for managing complexity, changes and uncertainties in manufacturing. *Engineering applications of artificial intelligence*, *16*(4), 277-291. | 0.2 | 0.9 | 17 |
| 12 | Monsifrot, A., Bodin, F., & Quiniou, R. (2002, September). A machine learning approach to automatic production of compiler heuristics. In *International conference on artificial intelligence: methodology, systems, and applications* (pp. 41-50). Springer, Berlin, Heidelberg. | 0.1 | 0.8 | 16 |
